# Dr. Pett

**Published:** 1823-02

**Authors:** 


### OBITUARY.

THE following particulars relative to the recent lamented death of Dr. PETT, of Clapton, we have obtained from Mr. TRAVERS, who politely drew them up at our request:—

At eight o'clock on the morning of Saturday, the 28th of December, Dr. Pett assisted a medical friend in examining the body of a lady who died of peritoneal inflammation after child-birth, on the Thursday preceding. At nine o'clock the same evening, being in his usual health and spirits, and while engaged at cards, he complained of an uneasiness in the middle finger of his right hand. On minute examination, a slight superficial wound was discovered. It was then touched with lunar caustic, and some time afterwards with a drop of strong sulphuric acid, neither of which applications was felt. On going to rest, he again applied the lunar caustic to the wound for the space of half a minute, which application was followed by pain, soon increasing to intensity; and, an hour after he had been in bed, he was attacked by a severe rigor, which continued more or less for three hours. The pain spread from the finger along the arm to the axilla, and was so agonizing as to lead him to observe, that he had never before known what pain was. The night he described as one of dreadful suffering, without intermission. In the morning, the expression of his countenance, and the altered manner of the man, struck his medical friend with instant alarm. The finger was white, and without sensation.

At twelve o'clock on Sunday, an incision was carried through the wound to the bone, which was not felt by Dr. Pett. In the course of this day, the arm became swollen, and the superficial absorbents conspicuous from inflammation. The pain affecting the arm extended to the axilla and pectoral region. The finger in a few hours became discoloured and gangrenous as far as the second joint, where suppuration of the soft parts afterwards took place. The high excitement of the nervous system, marked by a flushed countenance, a ferrety eye, vigilance, great anxiety, short and quick respiration, rapid and voluble speech, and unnaturally irritable manner, were accompanied by a very moderate acceleration of the pulse, which soon became intermittent, and then irregular.

On the morning of Tuesday, the arm had recovered its natural appearance; it was neither swollen nor painful, nor were any absorbent lines visible. A considerable effusion had taken place in the cellular substance of the axilla, and over the pectoral muscle: it was marked by an erythematous blush, was painful, and crepitated on pressure as in emphysema. The symptoms varied but little; but increased difficulty and hurry of respiration, and increased feebleness, were marked at each visit. He expressed a sense of confusion; but there was no further evidence of disturbed intellect than the deviation before adverted to from his natural characteristic calmness. The fulness at the axillary edge of the pectoral muscle being sensibly increased on the Wednesday morning, a lancet was pushed deeply into it, but only a bloody serum issued.

On the Wednesday evening, about six o'clock, he died, having survived the injury about 105 hours. The body was inspected on Friday, and no recent morbid appearance whatever was discovered in the chest or abdomen. The heart was unusually large, and its substance flabby. The internal structure of the liver had undergone considerable chronic degeneration. The head was not examined.

